# Evaluation of guideline adherence in enteral nutrition practices among ICU patients: a point prevalence study

**DOI:** 10.1136/bmjopen-2026-120718

**Published:** 2026-09-02

**Authors:** Betül Özgan, Yunus Emre Ayhan, Özge Özmen, Damla Sosyal, Güneş Eskidemir, Halime Yılmaz, Ahmet Çakır, Sıdıka Yetimoğlu, Ayşe Gül Koçoğlu Kınal, Muhammed Yunus Bektay, Hayrettin Daşkaya, Kazım Karaaslan, Funda Datlı Yakaryılmaz

**Affiliations:** 1 Department of Clinical Pharmacy, Faculty of Pharmacy, Fenerbahçe University, Ataşehir, Turkey; 2 Clinical Pharmacy Unit, Ministry of Health Istanbul Prof Dr Cemil Tascioglu City Hospital, Istanbul, Turkey; 3 Clinical Pharmacy Unit, Kartal Dr Lutfi Kirdar City Hospital, Istanbul, Turkey; 4 Clinical Pharmacy Unit, Basaksehir Cam and Sakura City Hospital, Istanbul, Turkey; 5 Department of Intensive Care Unıt, Gaziosmanpaşa Training and Research Hospital, Istanbul, Turkey; 6 Department of Clinical Pharmacy, Faculty of Pharmacy, Bezmialem Vakif University, Istanbul, Turkey; 7 Department of Clinical Pharmacy, Faculty of Pharmacy, Inonu University, Malatya, Turkey; 8 Intensive Care Unit, Dr. Siyami Ersek Thoracic and Cardiovascular Surgery Training and Research Hospital, Istanbul, Turkey; 9 Department of Clinical Pharmacy, Faculty of Pharmacy, Istanbul University-Cerrahpaşa, Avcılar, Turkey; 10 Department of Anesthesiology and Reanimation, Faculty of Medicine, Bezmialem Vakif University, Istanbul, Turkey; 11 Department of Geriatrics, Faculty of Medicine, İnönü University, Malatya, Turkey

**Keywords:** Observational Study, Adult intensive & critical care, Guideline Adherence, NUTRITION & DIETETICS

## Abstract

**Abstract:**

**Objectives:**

Malnutrition is a significant problem in critically ill patients and is associated with higher mortality, morbidity and healthcare costs. Enteral nutrition (EN), being more physiological than parenteral nutrition, helps reduce complications. This study evaluated how well EN practices in Turkish intensive care units (ICUs) align with current clinical nutrition guidelines.

**Design and setting:**

This multicentre, prospective, point-prevalence study was carried out in six ICUs in Türkiye.

**Participants:**

Adult patients (≥18 years) who had stayed in the ICU for at least 48 hours and had received EN for a minimum of 24 hours on either of the two prespecified point-prevalence dates (30 July and 30 August 2025) were eligible. All patients meeting these criteria were included.

**Interventions:**

No intervention was performed. Existing EN practices were evaluated against guideline-based recommendations.

**Primary outcome measures:**

EN practices were assessed according to initiation time, type of EN tube, method of EN administration, rate of achieving calorie and protein target, management of conditions requiring EN interruption and use of prokinetic agents. Guideline adherence was evaluated using a protocol based on European Society for Clinical Nutrition and Metabolism (ESPEN), American Society for Parenteral and Enteral Nutrition (ASPEN) and Turkish Society of Clinical Enteral and Parenteral Nutrition (KEPAN) recommendations.

**Results:**

98 patients were included; 60 patients (61.2%) were male, and the median age was 72 (IQR 59–79) years. EN was initiated within 48 hours in 72 patients (73.5%), while 13 (13.3%) delays lacked clinical justification. The median percentage of administered energy and protein compared with the targets was 87% (IQR 69.6%–100%) and 82% (IQR 64.9%–100%), respectively. However, only 27 patients (27.6%) achieved 100% of both energy and protein requirements. Failure to achieve nutritional targets was significantly associated with a higher number of comorbidities (p=0.007), higher body mass index (p=0.017) and renal dysfunction (p=0.041). EN continued despite contraindications in 14 patients (14.3%), who had significantly higher Acute Physiology and Chronic Health Evaluation II (APACHE II), Sequential Organ Failure Assessment (SOFA) and Nutrition Risk in Critically Ill (NUTRIC scores (all p<0.05).

**Conclusions:**

Among patients receiving EN, overall adherence to guideline recommendations was high. However, improvements are needed in achieving nutritional targets and optimising prokinetic use.

**STRENGTHS AND LIMITATIONS OF THIS STUDY**

- This multicentre point-prevalence study is the first national study to evaluate adherence to guideline-based enteral nutrition (EN) practices in adult intensive care units in Türkiye.
- The use of a standardised assessment protocol based on European Society for Clinical Nutrition and Metabolism (ESPEN), American Society for Parenteral and Enteral Nutrition (ASPEN) and Turkish Society of Clinical Enteral and Parenteral Nutrition (KEPAN) recommendations, together with independent evaluation by four experts, enhanced the consistency and validity of adherence assessments.
- Inclusion of multiple intensive care units with diverse clinical settings improved the representativeness of real-world EN practices across participating centres.
- The point-prevalence observational design, with data collected on only two study days, provides only a snapshot of clinical practice and precludes causal inference or assessment of long-term clinical outcomes.
- Unmeasured confounding factors and the lack of evaluation of organisational or educational barriers may have influenced guideline adherence.

## Introduction

 Management of critically ill patients in intensive care units (ICUs) requires a multidimensional approach that includes not only medical treatment but also nutrition support.^1^ Malnutrition affects 38–78% of ICU patients and has detrimental effects on clinical outcomes.^2^ It is associated with increased infectious complications, multiorgan dysfunction, prolonged hospitalisation, higher mortality and increased healthcare costs.^3–7^ Nutrition support therapy is widely implemented to maintain body mass, support metabolic functions and reduce complications.^4 8 9^ Among available methods, enteral nutrition (EN) stands out as being more physiological than parenteral nutrition (PN), supporting immune function, preserving gut integrity and reducing complications.^1 10–12^ Therefore, international guidelines recommend commencing EN early in all ICU patients without contraindications who are expected to stay longer than 48 hours.^1 12–14^ Despite these recommendations, existing studies show that EN practices often remain insufficient. Shin *et al* reported that nutrition support team (NST) consultations were mostly requested for PN recipients, whereas patients receiving EN had higher malnutrition risk and lower achievement of calorie and protein targets.^15^ Similarly, Hwang *et al* found significantly lower daily calorie and protein delivery in EN-only patients compared with PN or combined PN+EN recipients.^16^ Other studies have reported target achievement rates around 50–60%.^9 16–20^ Guidelines published by the Society of Critical Care Medicine (SCCM), American Society for Parenteral and Enteral Nutrition (ASPEN) and European Society for Clinical Nutrition and Metabolism (ESPEN) highlight the importance of early malnutrition risk assessment, timely initiation of EN, close monitoring of tolerance and avoiding unnecessary PN use.^1 13 21^ Studies have demonstrated that guideline adherence and NST involvement improve nutrition target achievement, reduce mortality, shorten length of stay, decrease costs and enhance overall outcomes.^6 19 20 22–26^

This study aims to evaluate the adherence of EN practices in critically ill ICU patients to national and international clinical guidelines using a point-prevalence methodology.

## Methods

### Study design and population

This multicentre, prospective, observational study used a repeated point-prevalence design and was conducted in the ICUs of six hospitals in Türkiye. Data were collected on two prespecified point-prevalence dates, 30 July and 30 August 2025. On each study date, all patients present in the participating ICUs were screened. Patients aged ≥18 years who had been hospitalised in the ICU for ≥48 hours and had received EN for ≥24 hours were eligible. All patients meeting these criteria were included without sampling. The study protocol, including data-collection procedures and ethical safeguards, was reviewed and approved by the institutional review boards of all participating centres. The study protocol was approved by the institutional review boards of all participating centres. Written informed consent was obtained from all participants or their legally authorised representatives. The study protocol was not prospectively registered because this was a multicentre observational point-prevalence study.

### Data collection

On the two designated days, EN-related data were extracted from electronic medical records and patient charts. All patients receiving nutrition support therapy on these days were screened, and those receiving EN were enrolled. Collected variables included demographic and clinical characteristics (age, sex, height, weight, body mass index (BMI)), admission diagnosis, admission date, comorbidities, length of ICU stay, renal and hepatic function, mechanical ventilation status, Sequential Organ Failure Assessment (SOFA), Glasgow Coma Scale (GCS), Nutrition Risk in Critically Ill (NUTRIC) score and Acute Physiology and Chronic Health Evaluation II (APACHE II). All parameters were routinely monitored clinical variables; no additional measurements were required for the study.

Nutrition-related variables included NST consultation status, indication for EN, EN initiation date, type of EN tube, targeted and delivered daily calorie/protein amounts, EN formula type, method of EN administration, intravenous glucose administration, gastric residual volume monitoring, fluid restriction, use of prokinetic agents and EN-related complications (aspiration, diarrhoea, constipation, nausea/vomiting, tube blockage, hyperglycaemia).

### Inclusion and exclusion criteria

Eligible participants were ICU patients ≥18 years old, hospitalised ≥48 hours and receiving EN for ≥24 hours. Patients with missing data, those receiving concurrent oral feeding and those receiving combined EN and PN were excluded.

### Evaluation of guideline adherence and assessment process

Adherence to EN guidelines was evaluated based on ESPEN, ASPEN and Turkish Society of Clinical Enteral and Parenteral Nutrition (KEPAN) recommendations, using a protocol developed jointly by two clinical pharmacists and two intensive care specialists (1, 13, 14, 22). The protocol development involved independent review of guidelines, resolution of disagreements through discussion and consensus via a Delphi-based process. The complete protocol is provided in online supplemental material 1.

After data collection, four experts independently reviewed EN practices for each patient using the same standardised protocol and classified them as ‘adherent’ or ‘non-adherent’. The evaluators were blinded to the participating ICU units during the assessment process, and all patient data were anonymised and coded to minimise potential bias.

The evaluation process consisted of two stages:

1. First round: Experts assessed adherence independently. Discrepant cases were identified for re-evaluation.
2. Second round: Experts reviewed discrepant assessments with knowledge of the group’s evaluations. Consensus was achieved for all cases. In cases of persistent disagreement, a joint meeting was held to finalise decisions.

### Primary outcome

The primary outcome was protocol adherence across six domains:

1. Timing of EN initiation.
2. Type of EN tube.
3. Method of EN administration.
4. Achievement of caloric and protein targets.
5. Management of EN-interrupting conditions.
6. Use of prokinetic agents.

### Study size

Because the study used a total-enumeration approach on two prespecified point-prevalence dates rather than sampling from a finite patient population, the study size was not determined using an a priori sample-size target. All patients present in the six participating ICUs on 30 July and 30 August 2025, were screened. Of the 260 screened patients, 98 met the prespecified eligibility criteria, and all eligible patients were included in the analysis. Thus, the study size was determined by the number of eligible patients available on the two study dates.

### Statistical analysis

All analyses were performed using IBM SPSS Statistics V.29.0. The distribution of continuous variables was assessed using the Kolmogorov-Smirnov test together with graphical and descriptive evaluations. Continuous variables were presented as mean and SD or median and IQR, as appropriate, whereas categorical variables were presented as numbers and percentages. Categorical variables were compared using the χ^2^ test or Fisher’s exact test, as appropriate. Continuous variables were compared between groups using Student’s t-test or the Mann-Whitney U test according to their distribution. All statistical tests were two-sided, and a p value of <0.05 was considered statistically significant.

This point prevalence study was reported in accordance with the Strengthening the Reporting of Observational Studies in Epidemiology (STROBE) guidelines for cross-sectional studies.

## Results

Across the two prespecified point-prevalence dates, 260 ICU patients were screened. Of these, 98 (37.7%) met the prespecified eligibility criteria and all eligible patients were included in the analysis. 60 patients (61.2%) were male; median age (IQR) was 72 (59–79). Median BMI was 25.7 kg/m² (23–27.9), with 22 patients (22.4%) classified as obese. The most common ICU admission diagnoses were respiratory failure (n=30, 30.6%), infection (n=14, 14.3%) and postoperative care (n=13, 13.3%). Median number of comorbidities was 2.5 (IQR 1–4). Patient recruitment was relatively balanced across the six participating ICUs, with centre contributions ranging from 10 to 20 patients (table 1).

**Table 1:** Sociodemographic and clinical data of the study patients

Variable	n=98
Participating intensive care unit, n (%)	
Intensive care unit 1	19 (19.4)
Intensive care unit 2	16 (16.3)
Intensive care unit 3	20 (20.4)
Intensive care unit 4	16 (16.3)
Intensive care unit 5	17 (17.3)
Intensive care unit 6	10 (10.2)
Age (years), median (IQR)	72 (59–79)
Sex, n (%)	
Male	60 (61.2)
Female	38 (38.8)
Body mass index (kg/m^2^), median (IQR)	25.7 (23–27.9)
Reason for hospitalisation, n (%)	
Respiratory failure	30 (30.6)
Infection	14 (14.3)
Postoperative care	13 (13.3)
Sepsis/septic shock	9 (9.2)
Other	32 (32.6)
Comorbidities, n (%)	
Hypertension	49 (50)
Diabetes mellitus	34 (34.7)
Coronary artery disease	34 (34.7)
Cancer	17 (17.3)
Heart failure	15 (15.3)
Cerebrovascular accident	13 (13.3)
Heart failure	12 (12.2)
Atrial fibrillation	12 (12.2)
Chronic kidney disease	12 (12.2)
Other	35 (35.7)
Number of comorbidities, median (IQR)	2.5 (1–4)
Length of stay (day), median (IQR)	15 (6–26)
APACHE score at admission, mean±SD	24±8
SOFA, mean±SD	6.3±3.4
NUTRIC score, median (IQR)	6 (4.75–7)
GCS score, median (IQR)	8 (4–10.25)
Sepsis/septic shock, n (%)	
Yes	40 (40.9)
No	58 (59.1)
Mechanical ventilation, n (%)	
Yes	85 (86.7)
No	13 (13.3)
Duration of mechanical ventilation (days), median (IQR)	15 (6–25)
Renal status, n (%)	
Normal	74 (75.5)
Acute kidney injury	15 (15.3)
Chronic kidney disease	9 (9.2)
Renal replacement therapy, n (%)	
Continuous haemodialysis	7 (7.1)
Intermittent haemodialysis	3 (3.1)
None	88 (89.8)
Hepatic status n (%)	
Normal	92 (93.9)
Acute injury	5 (5.1)
Chronic injury	1 (1)

APACHE, Acute Physiology and Chronic Health Evaluation; GCS, Glasgow Coma Scale; NUTRIC, Nutrition Risk in Critically Ill; SOFA, Sequential Organ Failure Assessment.

EN assessments were performed on median day 11 (IQR 4–23.25) of EN therapy. Nutrition support therapy consultation was requested for 30 patients (30.6%). The most common indication for EN was impaired consciousness (n=70, 71.4%). Median EN initiation day was 2 (IQR 1–3). The most frequently used EN formulas were diabetic (30.6%) and high-calorie formulas (22.4%). Hyperglycaemia (n=10, 10.2%) and constipation (n=9, 9.2%) were the most common EN-related complications. Median delivered calorie and protein amounts were 1425 kcal/day (IQR 1200–1685) and 75.1 g/day (IQR 50.3–96), respectively (table 2).

**Table 2:** Distribution of clinical findings related to enteral nutrition practices

Variable	n=98
Nutrition consultation, n (%)	
Nutrition support team	26 (26.5)
Dietitian	4 (4.1)
None	68 (69.4)
Indication for enteral nutrition, n (%)	
Altered consciousness	70 (71.4)
Dysphagia	16 (16.3)
Risk of malnutrition	12 (12.2)
Enteral nutrition initiation time (day), median (IQR)	2 (1–3)
Enteral nutrition product, n (%)	
Diabetic	30 (30.6)
High-calorie	22 (22.4)
Immunonutrition	18 (18.2)
Standard	15 (15.3)
Protein-rich	10 (10.2)
Semi-elemental	3 (3.1)
Complications associated with enteral nutrition, n (%)	
Hyperglycaemia	10 (10.2)
Constipation	9 (9.2)
Diarrhoea	6 (6.1)
Aspiration pneumonia	3 (3.1)
Nausea-vomiting	3 (3.1)
Tube occlusion	1 (1)
None	66 (67.3)
Daily calories (kcal/day), median (IQR)	1425 (1200–1685)
Daily protein (g/day), median (IQR)	75.1 (50.3–96)

**Figure 1:**
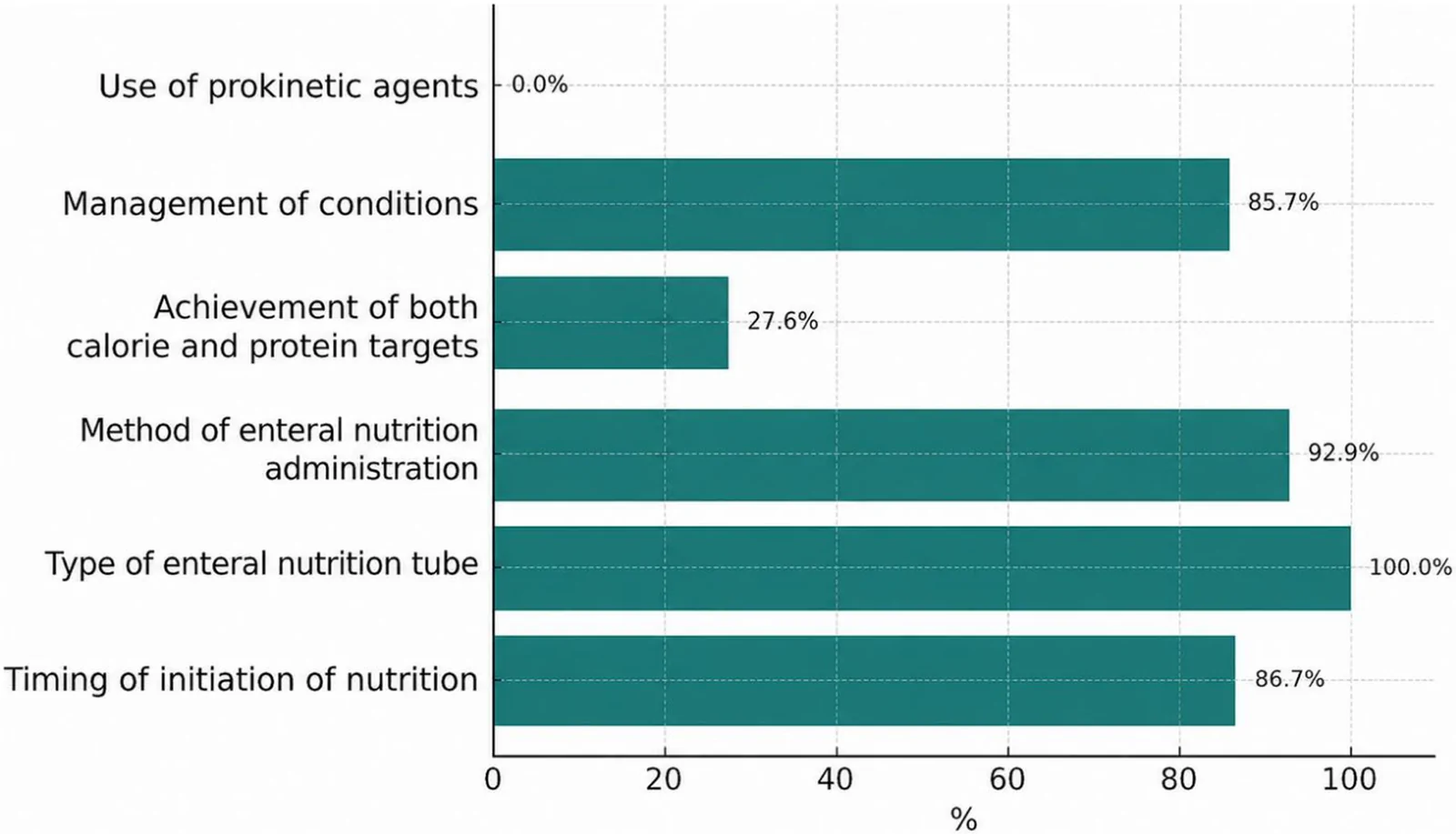
Evaluation of adherence to the enteral nutrition practice protocol.

EN was initiated within 48 hours in 72 patients (73.5%), while 26 (26.5%) experienced delays. No clinical justification for delay was found in 13 patients (13.3%). The median percentage of administered energy and protein compared with the targets was 87% (IQR 69.6–100) and 82% (IQR 64.9–100), respectively. These values represent the median proportion of individual target attainment across all patients. The distribution of patients according to predefined categories of energy and protein target attainment is presented in table 3. A total of 27 patients (27.6%) achieved 100% of both their administered energy and protein requirements. In addition, 18 patients (18.4%) exceeded the upper limit of their administered calorie target and 31 patients (31.6%) exceeded the upper limit of their administered protein target. Exceeding the calorie target was associated with NST consultation (p=0.02) and continuous EN administration (p=0.04), whereas exceeding the protein target was associated with high-calorie/high-protein formulas (p=0.032) and continuous EN administration (p<0.001). 14 patients (14.3%) had clinical conditions that required temporary EN interruption; however, EN was continued. Prokinetic agents were used in six patients (6.1%), all receiving intravenous metoclopramide (table 3). Adherence was highest for the type of EN tube and method of EN administration and lowest for target achievement and prokinetic agent use (figure 1).

**Table 3:** Evaluation of enteral nutrition protocol adherence

Variable	n=98
(1) Was nutrition (oral or enteral) initiated within the first 48 hours of the patient’s admission? n (%)	
Yes	72 (73.5)
(2) Were there any contraindications to initiating nutrition within the first 48 hours of admission? n (%)	
Yes	17 (17.3)
(3) Type of enteral nutrition tube, n (%)	
Nasogastric	90 (91.8)
Percutaneous endoscopic gastrostomy	8 (8.2)
(4) Method of enteral nutrition administration, n (%)	
Continuous infusion	76 (77.6)
Intermittent infusion	15 (15.3)
Bolus administration	7 (7.1)
(5) Rate of achieving caloric target, n (%)	
>100	18 (18.4)
%80≤ – %100	40 (40.8)
%60≤ – %80	24 (24.5)
%40≤ – %60	10 (10.2)
%20≤ – %40	5 (5.1)
<%20	1 (1)
(6) Rate of achieving protein target, n (%)	
>100	31 (31.6)
%80≤ – %100	24 (24.5)
%60≤ – %80	25 (25.5)
%40≤ – %60	14 (14.3)
%20≤ – %40	3 (3.1)
<%20	1 (1)
(7) Are there any situations where enteral nutrition initiation should be delayed?	
Uncontrolled shock	8 (8.2)
Uncontrolled hypoxaemia, hypercapnia or acidosis	3 (3.1)
Active upper gastrointestinal bleeding	1 (1)
Abdominal compartment syndrome	1 (1)
Obvious intestinal ischaemia	1 (1)
Gastric residual volume >500 mL every 6 hours	1 (1)
None	83 (84.7)
(8) Is there use of prokinetic agents? n (%)	
Yes	6 (6.1)

Nutrition support therapy consultation was significantly associated with appropriate EN initiation timing (p=0.046). Patients with appropriate initiation had lower SOFA scores (mean±SD 5.91±3.0 vs 9.46±4.3, p=0.001) and higher GCS scores (8 (5–11.5) vs 3 (3–8.5), p=0.009). Patients achieving full caloric/protein targets had fewer comorbidities (median (IQR) 1 (1–3) vs 3 (2–4), p=0.007) and lower BMI (median (IQR) 23.1 (21.1–27.7) vs 26.0 (23.9–31.2), p=0.017) and a lower prevalence of renal dysfunction (p=0.041). Patients within the 80–100% target range had significantly higher nutrition support therapy consultation rates (41.4% vs 15%, p=0.049) and lower obesity prevalence (13.8% vs 35%, p=0.03). Patients in whom EN was continued despite contraindications had significantly higher APACHE II (29±8 vs 23.33±7.8), SOFA (9.29±4.4 vs 5.89±3) and NUTRIC scores (7 (5–8) vs 6 (4–7)), all statistically significant (p<0.05). EN contraindications occurred significantly more often in renal dysfunction (62.5%) than in patients with normal renal function (6.8%) (p=0.001). No statistically significant differences were found between other demographic or clinical variables and the primary outcomes (p>0.05). Since the type of EN tube was appropriate in all patients and prokinetic agents were administered to only six patients (with all six cases being non-compliant with the protocol), no comparative analysis was performed. Detailed results are provided in online supplemental table 1.

## Discussion

This multicentre point-prevalence study provides comprehensive insights into EN practice patterns and guideline adherence in Turkish ICUs. Results highlight generally high levels of adherence but identify critical gaps in achieving nutritional targets and optimising prokinetic therapy.

In the literature, it has been demonstrated that initiating EN at the earliest possible time reduces mortality and lowers the risk of infection, particularly in ICU patients. In the present study, EN was found to be initiated in accordance with the protocol in the majority of patients. Furthermore, patients who received a nutrition consultation were more likely to have appropriate EN initiation timing than those who did not. Because the timing of nutrition support therapy consultation relative to EN initiation was not recorded, this association should not be interpreted as causal. Nevertheless, this finding suggests that nutrition consultation may be a marker of closer adherence to guideline-based nutritional care. The observation of higher SOFA scores and lower GCS scores in patients with delayed EN initiation—although these parameters do not constitute direct contraindications—may nevertheless have triggered a tendency among clinicians to postpone the procedure. Previous studies have similarly shown that protocol implementation or NST involvement shortens the time to EN initiation,^21–23 26–28^ supporting our findings. These findings are consistent with the observed association between nutrition support therapy consultation and appropriate EN initiation in the present study.

Guidelines recommend gastric access as the standard approach for initiating EN.^1 4^ Gastric access is more physiologically appropriate and easier to implement.^1^ For cases requiring EN for longer than 4 weeks, gastrostomy or jejunostomy routes are recommended.^1 14^ In the present study, gastric access was preferred for all patients, while gastrostomy was used in those requiring long-term feeding. Similarly, the literature reports that gastric access was selected in all patients or in more than 85% of patients across most studies.^20–22 29^

Current guidelines generally recommend continuous rather than bolus EN administration. Although continuous infusion has been associated with less diarrhoea, studies have reported comparable clinical outcomes between the two methods.^1 30 31^ Previous studies have also shown that continuous infusion is the preferred method of EN administration in the majority of ICU patients.^21 22 24^ Consistent with these findings, continuous infusion was used in most patients in the present study. Although no association was observed between the method of EN administration and EN-related complications continuous infusion was significantly associated with exceeding both calorie and protein targets, highlighting the importance of careful monitoring to avoid overfeeding.

Although only a small proportion of patients achieved 100% of the calorie and protein targets defined in the study protocol, most patients achieved more than 80% of these targets. This proportion is higher compared with values reported in the literature. In patient groups without protocol implementation or NST intervention, the target achievement rate typically ranges between 25% and 75%, with most studies reporting around 60%.^15–17 21 22 26 29 32^ Conversely, studies involving protocolised care or active NST participation report achievement rates exceeding 80%.^21 22 26 32^ The high achievement rate observed in the present study may be attributable to data collection on a single day. In long-term EN use, complications such as diarrhoea, vomiting and abdominal discomfort frequently lead to interruptions—often inappropriate—in EN delivery, making it difficult to meet calorie and protein targets. In this study, the majority of patients achieved 80–100% of both protein and calorie goals. These patients received significantly more nutrition consultations, and the higher consultation rate in the group that met targets aligns with the literature, highlighting its positive effect. Additionally, the number of comorbidities and BMI influenced the likelihood of meeting nutritional goals. Lower BMI and fewer comorbidities were associated with better target achievement, possibly because nutritional requirements are easier to meet and fewer clinical factors interfere with feeding. Similarly, patients with renal dysfunction were less likely to achieve nutritional targets, which may reflect greater illness severity and the complexity of nutritional management in this population. Consistently, obesity rates were significantly lower among patients who achieved 80–100% of their nutritional goals. Additionally, a considerable proportion of patients exceeded their administered calorie and protein targets. This finding suggests that, although achieving nutritional targets is important, regular reassessment of energy and protein requirements is also necessary to avoid overfeeding, particularly in patients receiving continuous EN or high-calorie/high-protein formulas.

Current guidelines recommend delaying EN in clinically unstable patients until life-threatening conditions, such as uncontrolled shock or severe hypoxaemia, are stabilised.^1 33 34^ In this study, EN was continued in some patients despite temporary contraindications, particularly uncontrolled shock. These patients had significantly higher APACHE, SOFA and NUTRIC scores, reflecting greater illness severity. Similarly, although renal dysfunction is not a contraindication to EN per se, its association with EN-interrupting conditions likely reflects disease severity rather than renal dysfunction itself. These findings highlight the importance of careful assessment and close monitoring of patients with clinical instability, particularly those with renal dysfunction or a high risk of malnutrition and mortality.

Prokinetic agents are recommended for ICU patients with gastric intolerance.^1^ As intravenous erythromycin is unavailable in Türkiye, intravenous metoclopramide was used in all patients requiring prokinetic therapy. However, none received treatment fully consistent with the protocol-defined dose and duration, indicating the need to improve adherence to guideline-based prokinetic use. Similar findings have been reported in previous Turkish ICU studies.^22 24^

### Strengths and limitations

This study has several limitations. First, its point-prevalence observational design provides only a snapshot of clinical practice and precludes causal inference. Data were collected on only two prespecified point-prevalence dates, which may not fully capture temporal variations in EN practices. Although all eligible patients available on these dates were included, the study population should be regarded as a census of the accessible population on the study dates rather than as a representative sample of all ICU patients receiving EN in Türkiye. Because of the cross-sectional point-prevalence design, the progressive advancement of EN over time could not be evaluated. Second, only six ICUs were included, potentially limiting the generalisability of the findings to other settings in Türkiye. Furthermore, only patients receiving EN were included, precluding the evaluation of patients who were eligible for EN but did not receive it. As patients were assessed at a single time point without longitudinal follow-up, total ICU length of stay and clinical outcomes, including mortality, could not be evaluated. In addition, point-prevalence sampling is inherently subject to length-time bias, as patients with longer ICU stays are more likely to be included. Unmeasured confounders, such as disease severity, staffing patterns and institutional differences, may also have influenced adherence assessments. Finally, organisational and educational factors affecting guideline adherence were not evaluated, and the large number of statistical comparisons may have increased the risk of type I error; therefore, these findings should be interpreted as exploratory.

Despite these limitations, the study has several notable strengths. Its multicentre design provides insight into EN practices across different ICU settings in Türkiye. To our knowledge, this is among the first national point-prevalence studies evaluating adherence to guideline-based EN practices in critically ill patients. The use of predefined assessment criteria based on ESPEN, ASPEN and KEPAN recommendations enhanced the consistency of evaluations, while inclusion of diverse ICU environments allowed capture of real-world variability in clinical practice.

## Conclusion

This multicentre point-prevalence study demonstrates a high level of guideline adherence among patients receiving EN in ICUs in Türkiye, while also identifying areas for improvement, particularly in achieving target nutritional goals and optimising prokinetic agent use. Although based on data collected on only two prespecified point-prevalence dates, the findings provide a basis for future longitudinal studies with longer observation periods. Failure to achieve nutritional targets was associated with patient-related factors, including comorbidity burden, BMI and renal dysfunction. These findings support the need for careful monitoring of nutritional target attainment in patients receiving EN.

## Supplementary material

10.1136/bmjopen-2026-120718 online supplemental file 1

10.1136/bmjopen-2026-120718 online supplemental table 1

## Data Availability

Data are available upon reasonable request.
