# Supplementary material for: Evaluation of guideline adherence in enteral nutrition practices among ICU patients: a point prevalence study
Source: BMJ Open. 2026 Sep 2;16(9):e120718. doi: 10.1136/bmjopen-2026-120718 (PMC13548273; doi:10.1136/bmjopen-2026-120718)
Supplement: Supplementary file 1 [file bmjopen-2026-120718.reviewer_comments.pdf]

PEER REVIEW HISTORY

BMJ Open publishes all reviews undertaken for accepted manuscripts. Reviewers are asked to complete a checklist review form and are provided with free text boxes to elaborate on their assessment. These free text comments are reproduced below.

ARTICLE DETAILS

Title (Provisional)

EVALUATION OF GUIDELINE ADHERENCE IN ENTERAL NUTRITION PRACTICES AMONG ICU PATIENTS: A POINT PREVALENCE STUDY

Authors

Özgan, Betül; Ayhan, Yunus Emre; Özmen, Özge; Sosyal, Damla; Eskidemir, Güneş; Yılmaz, Halime; Çakır , Ahmet; Yetimoğlu, Sıdıka; Koçoğlu Kınal, Ayşe Gül; Bektay, Muhammed Yunus; Daşkaya, Hayrettin; Karaaslan, Kazım; Datlı Yakaryılmaz, Funda

---

VERSION 1 - REVIEW

Reviewer **1**

Name **M. Bala, Malgorzata**

Affiliation **Jagiellonian University, Chair of Epidemiology and Preventive Medicine, Department of Hygiene and Dietetics**

Date **12-May-2026**

COI **None**

The authors submitted an interesting article, however additional information is required to fully understand the processes described.

Has the protocol of the study been registered?

The full protocol which was used for the assessment should be provided in the appendix so the readers are able to understand the process. Was the same protocol used across all scoring experts?

Did protocol contain clear information on when it should be scored as adhered and when it should score not adhered or subjective judgment of each expert was used? What was the process in the case of missing data? Was there any training of the experts or calibration exercise?

Could you provide more information on experts background?

The authors should discuss the limitation of the study listed at the beginning of the article and their impact in the results.

Since this is cross sectional study the authors should be careful in their conclusions and focus on associations not causal relationships.

Reviewer **2**

Name **Hiesmayr, Michael**

Affiliation **Medical University Vienna, Anaesthesia and General Intensive Care**

Date **22-May-2026**

COI **None**

The authors present the descriptive results of a point prevalence study in 6 Turkish ICU with a total of 130 available ICU beds on the practice of enteral nutrition (EN). 6 domains of adequacy of EN were assessed by 4 independent experts. The approach is interesting but several issues need to be clarified: 1. Abstract: The results indicate sometimes percentages without n and sometimes only n. Please be systematic. The results mirror the results in the text but contrast with the results in Table1. Example 87% and 82% energy / protein target reached in table target 80-100% reached 59 and 56%. Most probably the protocol states that > 60% of target is adequate. 2. Methods: The protocol developed by the 4 experts needs to be shown in order to understand what adequacy means. The sample size calculation is not clear. What means 5% EN rate and alpha 0.05 and 95% confidence? I think that it refers to the precision of estimates in observed proportions. For observed proportions this would be for 100 individuals at 50% CI95 [39.8%; 60.2%] and at 10% CI95 [4.9% to 17.6%]. This means that only relative large differences can be detected with the observed sample. From the 6 domains analyzed domain 2 appears to be already fulfilled by the selection of only patients with EN. If this is considered differently it would be clearer after disclosure of the protocol. Domain 6 (use of prokinetics) appears to have been applied to all patients regardless of gastric intolerance (that is quite low according to the table). For domain 3 duration a definition is missing and no information in Table 1. The duration information in Table 4 appears to indicate that there is an interaction with Bolus administration where the concept of duration does not really apply. For domain 4 meeting the target in energy and protein only categories up to 100% are considered. Has overshooting the target been considered? Domain 5 (contraindications within 48 hours) Not considering contraindications cannot be considered within a period but only at the timepoint of beginning please clarify. Did the duration yes/no Have the evaluators blinded to the 6 ICU contributing the patients. Statistics: The descriptive methods are well described but 92 statistical tests in Table 4 mandate the issue of multiple comparison to be addressed at least in limitations. Interaction between domains could show at least some interactions. Results: Table 1 demographics: please add outcome mortality / length of ICU stay to the table (of course mentioning length time bias typical for cross-sectional sampling). The proportion of adequacy for reaching the

target in abstract 87% and 82% and text differ from Table 3. (see above) . In Figure target is 27% probably the 27 without unit in abstract. But I feel it difficult to image 27% only meeting the target with 87 and 82 % in individual groups. The maximal separation is 0.87 x 0.82 equivalent to 71%. The interaction of target and length of stay in ICU at evaluation would be helpful. Table 4 is very difficult to read. The result for renal failure and insufficiency and target/interruptions might be confounded by severity of illness as it is not a typical contraindication for EN. Conclusion: The discussion could be much shorter. I could agree that EN is well done in this sample. For full understanding it would be important to know whether the most recent recommendation of a changing target over the first week in ICU was applied or not. In case of a fix target the results would be even more encouraging for Turkish practice (the generalisability from 6 ICUs to the whole country is to be discussed in limitations but the well presented data in Table 1 suggest a typical ICU population). A further limitation is the sampling (38% of patients) that does not allow to state how many patient did not get EN despite being appropriate candidates.

VERSION 1 - AUTHOR RESPONSE

Reviewer: 1 Dr. Malgorzata M. Bala, Jagiellonian University Comments to the Author:
The authors submitted an interesting article, however additional information is required to fully understand the processes described.

1. Has the protocol of the study been registered?

   Thank you for this valuable comment. A statement clarifying that the study was conducted according to a predefined protocol with ethical approval obtained before data collection, despite not being prospectively registered, has been added to the Study Design and Population subsection of the Methods (page 3, lines 131-132).

2. The full protocol which was used for the assessment should be provided in the appendix so the readers are able to understand the process.

   Thank you for this valuable comment. The full assessment protocol has been added as a supplementary appendix to the manuscript.

3. Was the same protocol used across all scoring experts?

   Thank you for this valuable comment. The manuscript has been revised to clarify that the same standardized protocol was used by four independent experts. This information has been added to the Evaluation of Guideline Adherence and Assessment Process subsection of the Methods (page 4, lines 165-166).

4. Did protocol contain clear information on when it should be scored as adhered and when it should score not adhered or subjective judgment of each expert was used?

   Thank you for this valuable comment. The full protocol will be provided as a supplementary appendix to clarify the evaluation process in detail. The protocol defines

optimal practice criteria for each domain. Four independent experts assessed each patient's data in relation to their clinical context and classified practices accordingly. The evaluation was conducted in two rounds, during which discrepant cases were re-reviewed and a consensus decision was reached.

5. What was the process in the case of missing data?

   Thank you for this valuable comment. There were no missing data in the study, as all required variables were fully available for all included patients.

6. Was there any training of the experts or calibration exercise? Could you provide more information on experts background?

   Thank you for this valuable comment. The expert panel consisted of two clinical pharmacists and two intensive care specialists, all of whom had clinical experience in intensive care settings and nutrition support practices, including enteral nutrition management in critically ill patients.
   Although a separate formal training or calibration exercise was not conducted, the evaluation protocol was developed through a structured process involving independent review of ESPEN, ASPEN, and KEPAN guidelines, followed by iterative discussions and consensus using a Delphi-based approach. This ensured a shared understanding of the assessment criteria prior to application. The same expert panel independently applied the finalized protocol to assess adherence to enteral nutrition guidelines.

7. The authors should discuss the limitation of the study listed at the beginning of the article and their impact in the results. Since this is cross sectional study the authors should be careful in their conclusions and focus on associations not causal relationships.

   Thank you for this valuable comment. We have revised the Discussion section to more explicitly address the study's limitations and their potential impact on the interpretation of the findings, as well as to further highlight the main strengths of the study (pages 9, lines 412-413)

Reviewer: 2 Prof. Michael Hiesmayr, Medical University Vienna, Medical University of Vienna Comments to the Author: The authors present the descriptive results of a point prevalence study in 6 Turkish ICU with a total of 130 available ICU beds on the practice of enteral nutrition (EN). 6 domains of adequacy of EN were assessed by 4 independent experts. The approach is interesting but several issues need to be clarified:

1. The results indicate sometimes percentages without n and sometimes only n. Please be systematic.

   Thank you for this valuable comment. The Abstract has been revised to ensure consistent reporting of results throughout, with categorical variables systematically presented as absolute numbers and percentages (page 1, Abstract)

2. The results mirror the results in the text but contrast with the results in Table1. Example 87% and 82% energy / protein target reached in table target 80-100% reached 59 and 56%. Most probably the protocol states that > 60% of target is adequate.

Thank you for this important comment. We agree that the original wording may have been unclear. The Abstract has been revised to clarify that the reported values (87% and 82%) represent the median proportion of prescribed energy and protein targets achieved, rather than the proportion of patients reaching those targets. We have also clarified the distinction between these summary measures and the proportion of patients achieving 100% of both energy and protein requirements (page 1, lines 19-21). For clarification, Table 3 presents the distribution of patients across predefined categories of energy and protein target attainment, whereas the values of 87% and 82% reflect the median percentage of target achievement across individual patients.

3. The protocol developed by the 4 experts needs to be shown in order to understand what adequacy means.

   Thank you for this valuable suggestion. To improve transparency and facilitate interpretation of the assessment criteria, the evaluation protocol developed by the four experts has been included as a supplementary appendix in the revised manuscript.

4. The sample size calculation is not clear. What means 5% EN rate and alpha 0.05 and 95% confidence? I think that it refers to the precision of estimates in observed proportions. For observed proportions this would be for 100 individuals at 50% CI95 [39.8%; 60.2%] and at 10% CI95 [4.9% to 17.6%]. This means that only relative large differences can be detected with the observed sample.

   Thank you for this important comment. We agree that the original description of the sample size calculation was unclear. The Methods section has been revised to clearly describe the parameters used for the calculation, including the expected population proportion (20%), 5% margin of error, and 95% confidence level. This revision clarifies that the sample size calculation was based on estimating a population proportion with a specified level of precision. These revisions have been made on page 4, lines 184-186 and page 5 lines 191-193.

5. From the 6 domains analyzed domain 2 appears to be already fulfilled by the selection of only patients with EN. If this is considered differently it would be clearer after disclosure of the protocol.

   Thank you for this valuable comment. To improve transparency, the evaluation protocol has been included as a supplementary appendix in the revised manuscript. In addition, to ensure consistent terminology throughout the manuscript, including the tables, the term **"Type of enteral nutrition tube"** has been used consistently to describe Domain 2 (page 1, line 13/ page 3, line 144/ page 6, line 253/ page 6-7, lines 267, 278).

6. Domain 6 (use of prokinetics) appears to have been applied to all patients regardless of gastric intolerance (that is quite low according to the table).

   Thank you for this valuable comment. Our audit revealed that prokinetic agents were administered to only six patients in our cohort. These cases were considered non-adherent to the protocol because at least one of the following applied: there was no documented evidence of gastric intolerance [there was no other valid clinical indication for prokinetic use (e.g., nausea or vomiting)], the prescribed dose did not comply with the protocol, or the duration of therapy exceeded the recommended limits. To clear up any misunderstanding,

we have thoroughly revised the corresponding statement in the Results section (page 7, lines 278-279) to explicitly state that a comparative analysis was omitted because enteral nutrition tube types were appropriate for all patients and prokinetic use was limited to these 6 entirely non-compliant cases.

7. For domain 3 duration a definition is missing and no information in Table 1. The duration information in Table 4 appears to indicate that there is an interaction with Bolus administration where the concept of duration does not really apply.

   Thank you for this insightful comment. We agree that the term 'administration duration' was ambiguous and clinically inapplicable to patients receiving bolus enteral nutrition. To address this issue and ensure conceptual clarity, we have removed the phrase 'administration duration' from the entire manuscript and tables. Instead, we have consistently adopted the term **'Method of EN administration'** (Domain 3) across the text and tables to accurately reflect whether nutrition was delivered via continuous, intermittent, or bolus methods. As noted in a previous response, the full evaluation protocol has also been included as a supplementary appendix, which we believe will provide further clarification on the assessment criteria for this domain (page 1, line 13-14/ page 4 line 179/ page 6, line 253).

   Regarding Table 1, we would like to clarify that this table was strictly designed to present the baseline clinical and socio-demographic characteristics of the patients. For this reason, specific nutritional protocol data, such as the method of EN administration, were intentionally omitted from Table 1 and are instead detailed in the subsequent tables focusing on nutritional practices.

8. For domain 4 meeting the target in energy and protein only categories up to 100% are considered. Has overshooting the target been considered?

   Thank you for this insightful comment, which helped us identify an important aspect that had not been explicitly reported in the original manuscript. Because failure to achieve prescribed calorie and protein targets is more commonly encountered in ICU patients and its adverse clinical consequences are well established, our study primarily focused on undernutrition and target attainment. However, as you pointed out, exceeding prescribed nutritional targets is also an important issue that warrants evaluation. In response, we evaluated patients who exceeded the upper limits of their prescribed calorie and protein targets. We identified 18 (18.4%) patients who exceeded the calorie target and 31 (31.6%) patients who exceeded the protein target, and these data have now been added to Table 3 (page 6, lines 245–250).

   We also performed additional analyses to explore factors associated with target overshooting. Exceeding the calorie target was significantly associated with nutrition support therapy consultation ($p=0.02$) and the method of enteral nutrition administration ($p=0.04$), with continuous infusion being more common among patients exceeding the target. Exceeding the protein target was significantly associated with the type of enteral nutrition product ($p=0.032$), with high-calorie/high-protein formulas being used more frequently, and with the method of enteral nutrition administration ($p<0.001$), again reflecting a higher proportion of continuous infusion. These findings have been incorporated into the Results section (page 6, lines 245-250).

We have expanded the Discussion to address the clinical implications of exceeding prescribed calorie and protein targets. We now emphasize the importance of regular reassessment of nutritional requirements during EN therapy to avoid overfeeding, particularly in patients receiving continuous enteral nutrition or high-calorie/high-protein formulas (page 8, lines 334-336 and lines 354-358).

9. Domain 5 (contraindications within 48 hours) Not considering contraindications cannot be considered within a period but only at the timepoint of beginning please clarify.

   Thank you for the opportunity to clarify this point. We would like to state that Domain 5 does not evaluate a retrospective 48-hour period. Rather, it assesses the presence of contraindications strictly *on the specific day of data collection (the audit day)* to evaluate whether administering EN on that cross-sectional study day was appropriate based on the patient's immediate clinical status at that specific timepoint.

   In contrast, the 48-hour window is evaluated under Domain 1, which assesses whether a delay in initiating EN beyond the first 48 hours of admission was clinically justified due to a contraindication present *during that initial period*. Because the current definitions in the manuscript already isolate Domain 5 to the specific day of data collection and Domain 1 to the initial initiation window, no further changes were made to the text.

10. Did the duration yes/no Have the evaluators blinded to the 6 ICU contributing the patients.

    Thank you for this important point. The evaluators were blinded to the participating ICU units during the assessment. Patient data were anonymized and coded, and no information regarding the ICU of origin was available to the experts in order to minimize potential bias in the evaluation process. We have clarified and updated the Methods section to explicitly reflect the blinding procedure (page 4, lines 166-168).

11. The descriptive methods are well described but 92 statistical tests in Table 4 mandate the issue of multiple comparison to be addressed at least in limitations.

    Thank you for this important comment. We acknowledge that performing multiple statistical tests (n=92 in Table 4) may increase the risk of type I error due to multiple comparisons. This limitation has now been explicitly added to the Limitations section of the manuscript (page 9, lines 423-425)

12. Interaction between domains could show at least some interactions.

    Thank you for this valuable suggestion. In response, we explored the relationships between the six domains of enteral nutrition practice by performing additional comparative analyses. However, no statistically significant associations were identified between the domains. As these analyses did not provide additional clinically meaningful findings, they were not incorporated into the main manuscript

13. Table 1 demographics: please add outcome mortality / length of ICU stay to the table (of course mentioning length time bias typical for cross-sectional sampling).

Thank you for this valuable suggestion. Because our study utilized a point-prevalence (cross-sectional) design, patient data were collected strictly within the specific study days without any subsequent longitudinal follow-up. For this reason, the total length of ICU stay and clinical outcome data (such as mortality) were not recorded and unfortunately could not be added to Table 1. Instead, as presented in Table 1, we reported the length of ICU stay *up to the specific day of data collection*.

We completely agree with the reviewer regarding the inherent risk of **length time bias** typical for cross-sectional sampling—where patients with longer ICU stays are more likely to be captured on the point-prevalence day. To acknowledge this methodological characteristic and clear up any ambiguity, we have added a statement regarding this limitation to the Discussion section (Page 9, Lines 417-419)

14. The proportion of adequacy for reaching the target in abstract 87% and 82% and text differ from Table 3. (see above).

We thank the reviewer for this observation. The percentages reported in the Results section (87% for energy and 82% for protein) represent the median proportion of the prescribed energy and protein targets achieved by individual patients, rather than the proportion of patients reaching a predefined target range. In contrast, Table 3 presents the distribution of patients according to predefined categories of target attainment (e.g., 80–100%, 60–80%, 40–60%, and <20% of target achieved).

We agree that this distinction may not have been sufficiently clear in the original manuscript. Therefore, we have revised the Results section to explicitly clarify how these percentages were calculated and to avoid potential misinterpretation (page 6, lines 240-241).

15. In Figure target is 27% probably the 27 without unit in abstract. But I feel it difficult to image 27% only meeting the target with 87 and 82 % in individual groups. The maximal separation is 0.87 x 0.82 equivalent to 71%.

Thank you for this important observation. The 27% shown in the figure represents the proportion of patients who simultaneously achieved both calorie and protein targets (i.e., 100% achievement of both targets). This distinction has been clarified in the Results section, (page 6, lines 244-245) and the figure has been revised to explicitly state "achievement of both calorie and protein targets" to avoid misinterpretation.

16. The interaction of target and length of stay in ICU at evaluation would be helpful.

Thank you for this valuable suggestion. This analysis was performed in the original manuscript. The association between achievement of both calorie and protein targets and ICU length of stay up to the day of assessment was evaluated and is presented in Table 4. No statistically significant association was observed ($p = 0.148$).

17. Table 4 is very difficult to read.

Thank you for this helpful suggestion. To improve readability, we have revised the Results section by describing all statistically significant findings in the text. In addition, Table 4

has been moved to the Supplementary Material, as it primarily contains detailed comparative analyses and numerous non-significant results.

18. The result for renal failure and insufficiency and target/interruptions might be confounded by severity of illness as it is not a typical contraindication for EN.

    Thank you for this important observation. We agree that renal failure or insufficiency is not, by itself, a typical contraindication to enteral nutrition. However, the significant association observed in our study was between renal dysfunction and the domain "Management of EN-interrupting conditions," rather than renal dysfunction being considered a contraindication itself. We believe this finding likely reflects the greater severity of illness among patients with renal dysfunction, who are more likely to develop conditions requiring interruption or modification of enteral nutrition. This interpretation is consistent with our discussion, where we state that renal dysfunction is associated with adverse outcomes in critically ill patients, making the higher prevalence of EN-interrupting conditions in this population clinically plausible. To further clarify this point, we have revised the Discussion section (page 9, lines 400-402)

19. **Conclusion:** The discussion could be much shorter. I could agree that EN is well done in this sample.

    Thank you for this helpful suggestion. In response, we have substantially shortened the Discussion by removing repetitive background information and focusing on the key findings and their clinical implications, while preserving the main messages of the study.

20. For full understanding it would be important to know whether the most recent recommendation of a changing target over the first week in ICU was applied or not.

    Thank you for this important comment. Yes, the evaluation protocol incorporated the current recommendations regarding the progressive achievement of energy targets during the first week of ICU stay. Specifically, hypocaloric feeding (20–25 kcal/kg/day) was considered appropriate during the acute and initial phases of critical illness, whereas isocaloric feeding (25–30 kcal/kg/day) was expected after days 7, in accordance with current guidelines. Protein targets were also adapted according to renal function and the use of renal replacement therapy. To make this process more transparent, the detailed evaluation protocol has been provided as an appendix.

21. In case of a fix target the results would be even more encouraging for Turkish practice (the generalisability from 6 ICUs to the whole country is to be discussed in limitations but the well presented data in Table 1 suggest a typical ICU population).

    Thank you for this thoughtful comment. We agree that applying the current recommendations of progressively increasing nutritional targets during the first week of ICU stay provides a more stringent assessment than using a fixed target for all patients. As described above, these recommendations were incorporated into our evaluation protocol.

    We also agree that the inclusion of only six ICUs may limit the generalisability of our findings. Accordingly, this limitation has now been explicitly added to the Limitations section of the manuscript (page 9, lines 414-416).

22. A further limitation is the sampling (38% of patients) that does not allow to state how many patient did not get EN despite being appropriate candidates.

    Than you for this important comment. We agree that, because only patients receiving enteral nutrition were included in the study, we were unable to identify patients who may have been appropriate candidates for enteral nutrition but did not receive it. Consequently, our findings reflect adherence to enteral nutrition practices among patients who received EN rather than the appropriateness of EN initiation in the entire ICU population. This limitation has now been added to the Limitations section of the manuscript (page 9, lines 416-417)

VERSION 2 - REVIEW

Reviewer **1**

Name **M. Bala, Malgorzata**

Affiliation **Jagiellonian University, Chair of Epidemiology and Preventive Medicine, Department of Hygiene and Dietetics**

Date **27-Jul-2026**

COI

Thank you. My comments were addressed properly.

Reviewer **2**

Name **Hiesmayr, Michael**

Affiliation **Medical University Vienna, Anaesthesia and General Intensive Care**

Date **28-Jul-2026**

COI

The authors have extensively revised the manuscript according to the recommendations.

There are several issues that need to be addressed:

Abstract:

The sentence:" The median proportion of prescribed energy and protein targets achieved was 87% (IQR 69.6-100) and 82% (IQR 64.9-100), respectively" may be correctly rephrased to " The median percentage of administered energy and protein compared with the targets was 87% (IQR 69.6-100) and 82% (IQR 64.9-100), respectively."

I think that it would be informative that most deviations in the target were in patients with abnormal kidney function.

The conclusion should really focus on patients receiving EN are treated according to guidelines. EN practices appears very large since the study does not have any information about patients that should receive EN but did not receive it.

The sentence : "Standardizing nutritional care and strengthening multidisciplinary collaboration may enhance clinical outcomes." should be erased because this is not part of the research presented.

There is a concern about the nutrition variable " NST consultation status". If this means that the patient was seen between ICU admission and the data collection ICU day (14 days) by the NST team any association or even "causal association" between starting timepoint of EN and consultation status is erroneous since the consultation may well have happened after the start of EN. As only 30/98 had a consultation it does not appear to be institutional practice to consult all patients before starting EN. Of course the consultation status appears to be relevant (associated with) for not meeting the target.

Another issue is the representativeness of the ICUs. The contribution of individual ICUs to the sample is indicated. It would be important to know whether few ICUs were major contributor.

The sample size calculation is still probably not meaningful. The authors state " single population proportion formula" with an "expected proportion of 20%, 5% margin of error and 95% confidence level". How do the now 135 beds from previously 130 beds enter in the calculation? In a one-sample design you can only define precision of the estimate based on sample size. Please explain what is the "expected proportion" and why you refer to 95% confidence level if all data are presented as median with interquartile range.

I have tried to calculate a sample size with STATA 19.0 and could only obtain a total of 88 patients if 2 groups with proportion 100% and 80% were assessed. But this is not the study design. Unless the sample size calculation can be made transparent it should not be given. It suggest a "scientific approach" that is not really convincing since the sample size nearly doubled from the first version.

The provision of the agreed guideline summary is helpful for understanding. The step from hypocaloric to normocaloric is not really what actual guidelines understand as progressive nutrition. The buildup of nutrition could not be studied in the studied sample since only a quarter of patients was studied before day 7 due to the cross-sectional sampling. The authors could state that the progressive build up could not be studied.

The additional table 1 is better readable but the item " Rate of achieving caloric target (%), Median (IQR)" should be replaced by " Percentage of administered calories / protein compared with target" . Rate is clearly inappropriate.

In all the text "prescribed" should be replaced by "administered" since the prescription practice was not the research question and independently collected if I understand the protocol well.

VERSION 2 - AUTHOR RESPONSE

Reviewer: 2

1. **The sentence:"The median proportion of prescribed energy and protein targets achieved was 87% (IQR 69.6-100) and 82% (IQR 64.9-100), respectively" may be correctly rephrased to " The median percentage of administered energy and protein compared with the targets was 87% (IQR 69.6-100) and 82% (IQR 64.9-100), respectively."**

Response: Thank you for this helpful suggestion. We agree that the term *administered* more accurately reflects the variable evaluated in our study. Accordingly, we have revised the wording in both the **Abstract** and the **Results** sections to read: *"The median percentage of administered energy and protein compared with the targets..."* throughout the manuscript (page 1, lines 20,21/ page 6, lines 212-214).

2. **I think that it would be informative that most deviations in the target were in patients with abnormal kidney function.**

Response: Thank you for this valuable suggestion. We agree that this finding provides important clinical context. Accordingly, we revised both the **Abstract** and the **Results** sections to include the significant association between failure to achieve nutritional targets and renal dysfunction. We also added the significant associations with a higher number of comorbidities and higher BMI. Furthermore, we revised the **Discussion** section to discuss these findings and their potential clinical implications (page 1, lines 23-24/ page 7 line 240/ page 9, lines 315-317)

3. **The conclusion should really focus on patients receiving EN are treated according to guidelines. EN practices appears very large since the study does not have any information about patients that should receive EN but did not receive it.**

Response: Thank you for this important comment. We agree that our study evaluated only patients who were already receiving enteral nutrition and did not include patients who were eligible for EN but did not receive it. Accordingly, we have revised the **Conclusion** section to clarify that our findings apply specifically to patients receiving EN. We also removed statements that extended beyond the scope of the present study and revised the conclusion to focus on the main findings supported by our results (page 11, lines 368-375).

4. **The sentence : "Standardizing nutritional care and strengthening multidisciplinary collaboration may enhance clinical outcomes." should be erased because this is not part of the research presented.**

Response: Thank you for this comment. As suggested, we removed the statement regarding the potential benefits of standardizing nutritional care and strengthening multidisciplinary collaboration, as it was beyond the scope of the present study. The **Conclusion** in the Abstract has been revised accordingly to focus only on the findings directly supported by our results (page 1, lines 26-28).

1. **There is a concern about the nutrition variable " NST consultation status". If this means that the patient was seen between ICU admission and the data collection ICU day (14 days) by the NST team any association or even "causal association" between starting timepoint of EN and consultation status is erroneous since the consultation may well have happened after the start of EN. As only 30/98 had a consultation it does not appear to be institutional practice to consult all patients before starting EN. Of course the consultation status appears to be relevant (associated with) for not meeting the target.**

Response: Thank you for this important observation. We agree that the timing of nutrition support therapy consultation relative to EN initiation was not available in our dataset; therefore, a causal relationship cannot be inferred. Accordingly, we revised both the **Results** and **Discussion** sections to describe this finding as an **association** rather than implying causality. We also added a statement acknowledging that the observed association should be interpreted with caution because the consultation may have occurred after EN initiation (page 7 lines 235-236/ page 8, lines 263-267).

2. **Another issue is the representativeness of the ICUs. The contribution of individual ICUs to the sample is indicated. It would be important to know whether few ICUs were major contributor.**

Response: Thank you for this valuable comment. To clarify the representativeness of the participating centers, we have added the distribution of patients across the six participating ICUs to **Table 1** and referred to this information in the **Results** section. Patient recruitment was relatively balanced across the participating ICUs, indicating that the study findings were not driven by a small number of centers (page 6, lines 201-203/ page 14).

3. **The sample size calculation is still probably not meaningful. The authors state " single population proportion formula" with an "expected proportion of 20%, 5% margin of error and 95% confidence level". How do the now 135 beds from previously 130 beds enter in the calculation? In a one-sample design you can only define precision of the estimate based on sample size. Please explain what is the "expected proportion" and why you refer to 95% confidence level if all data are presented as median with interquartile range. I have tried to calculate a sample size with STATA 19.0 and could only obtain a total of 88 patients if 2 groups with proportion 100% and 80% were assessed. But this is not the study design. Unless the sample size calculation can be made transparent it should not be given. It suggest a "scientific approach" that is not really convincing since the sample size nearly doubled from the first version.**

Response: Thank you for this thoughtful comment. We agree that an a priori sample size calculation was not appropriate for the design of our study. Accordingly, we have removed the sample size calculation from the manuscript and replaced the **"Sample Size"** subsection with a **"Study Size"** subsection. We now clarify that this was a point-prevalence study using a total-enumeration approach on two prespecified study dates. Therefore, no a priori sample size target was defined. Instead, all patients present in the six participating ICUs on the two study dates were screened, and all patients meeting the predefined eligibility criteria were included in the analysis. We have also revised the relevant

statements throughout the manuscript to ensure consistency with this methodological approach (page 1, lines 9-12/ page 3, lines 85-90/ page 5, lines 150-156/ page 5, lines 158-165/ page 6, lines 196-198/ page 10 lines 341-345)

4. **The provision of the agreed guideline summary is helpful for understanding. The step from hypocaloric to normocaloric is not really what actual guidelines understand as progressive nutrition. The buildup of nutrition could not be studied in the studied sample since only a quarter of patients was studied before day 7 due to the cross-sectional sampling. The authors could state that the progressive build up could not be studied.**

Response: Thank you for this helpful comment. We agree that the concept of progressive advancement of enteral nutrition is not fully reflected by a simple transition from hypocaloric to normocaloric feeding. we have acknowledged in the **Limitations** section that, because of the cross-sectional point-prevalence design, the progressive advancement of enteral nutrition over time could not be evaluated (page 10, lines345-347).

5. **The additional table 1 is better readable but the item " Rate of achieving caloric target (%), Median (IQR)" should be replaced by " Percentage of administered calories / protein compared with target" . Rate is clearly inappropriate.**

Response: Thank you for this helpful suggestion. Accordingly, we revised the terminology in **Supplementary Table 1** to *"Percentage of administered calories/protein compared with target, Median (IQR)"* for consistency with the manuscript and to more accurately reflect the reported variable.

6. **In all the text "prescribed" should be replaced by "administered" since the prescription practice was not the research question and independently collected if I understand the protocol well.**

Response: Thank you for this important comment. Accordingly, we have replaced *"prescribed"* with *"administered"* throughout the manuscript (page 6, lines 217, 218, 219).

VERSION 3 - REVIEW

**Reviewer**	**2**
**Name**	**Hiesmayr, Michael**
**Affiliation**	**Medical University Vienna, Anaesthesia and General Intensive Care**
**Date**	**14-Aug-2026**
**COI**	

Authors have answered to all comments.
