# Supplementary material for: Evaluation of guideline adherence in enteral nutrition practices among ICU patients: a point prevalence study
Source: BMJ Open. 2026 Sep 2;16(9):e120718. doi: 10.1136/bmjopen-2026-120718 (PMC13548273; doi:10.1136/bmjopen-2026-120718)
Supplement: Supplementary file 2 [file bmjopen-16-9-s001.docx]

**ENTERAL NUTRITION PRACTICE PROTOCOL IN THE INTENSIVE CARE UNIT**

**1. Timing of initiation of nutrition**

- Medical nutrition therapy should be considered for all patients admitted to the intensive care ünit (ICU), particularly those with a stay exceeding 48 hours¹.
- If oral intake is not feasible, early initiation of enteral nutrition (EN) within 48 hours should be preferred over delaying EN in critically ill adult patients¹.
- If oral intake is not feasible, early EN (within 48 hours) should be initiated rather than early parenteral nutrition (PN) in critically ill adult patients¹.

**2. Type of EN tube**

- Gastric access should be employed as the standard approach for initiating EN¹.
- If the anticipated duration is shorter than four weeks, feeding should be provided via nasally inserted feeding tubes; if longer, gastrostomy or jejunostomy routes should be preferred².
- In patients with gastric feeding intolerance unresponsive to prokinetic agents, postpyloric feeding should be employed¹.

**3. Method of EN administration**

- Continuous EN should be used instead of bolus EN¹.

**4. Achievement of target calorie and protein targets**

- A caloric intake of 20–30 kcal/kg/day in critically ill patients reasonably meets the average energy requirements and may be used when indirect calorimetry is not available³.
- The energy target should be hypocaloric (20–25 kcal/kg/day) during the acute and initial phases of illness, and isocaloric (25–30 kcal/kg/day) after days 7¹^,^²^,^⁴^,^⁵.
- For patients receiving renal replacement therapy (RRT), the additional calories supplied via dialysis solutions in the form of citrate, lactate, and glucose should be included in the calculation of total daily energy².
- **Protein requirements in acute kidney injury (AKI), AKI superimposed on chronic kidney disease (CKD), and CKD in acute/critically ill patients³:**
  - Not receiving RRT: Start with 1 g/kg/day; if tolerated, increase up to 1.3 g/kg/day.
  - Receiving intermittent RRT: 1.3–1.5 g/kg/day.
  - Receiving continuous renal replacement therapy (CRRT): 1.5–1.7 g/kg/day.
- Pre-admission body weight or normal body weight may be preferred over ideal body weight.
- In obese patients, if indirect calorimetry is not available, energy intake may be based on adjusted body weight, and protein intake may be prescribed as 1.3 g/kg “adjusted body weight”/day¹.

**5. Management of EN-interrupting conditions**

**EN should be delayed under the following conditions¹:**

- Uncontrolled shock, when hemodynamic and tissue perfusion targets cannot be achieved*.
- Uncontrolled, life-threatening hypoxemia, hypercapnia, or acidosis.
- Active upper gastrointestinal bleeding.
- Overt intestinal ischemia.
- In patients with intestinal fistula, if reliable feeding access cannot be secured distal to the fistula.
- Abdominal compartment syndrome.
- Gastric aspirate volume (gastric residual volume) exceeding 500 mL/6 hours.

*High-dose vasopressor requirement (e.g., norepinephrine >1 mcg/kg/min), hyperlactatemia, or other signs of end-organ hypoperfusion.

**6. Use of prokinetic agents**

- In critically ill patients with gastric feeding intolerance, intravenous erythromycin (3–7 mg/kg/day) should be used as the first-line prokinetic therapy. Alternatively, intravenous metoclopramide (3 × 10 mg) or a combination of metoclopramide and erythromycin may be administered¹.
- The efficacy of erythromycin and other prokinetic agents declines to one-third after 72 hours and they should be discontinued after three days¹.

**Other Conditions**

- A comprehensive clinical assessment should be performed to evaluate malnutrition in ICU patients¹.
- To prevent overfeeding, full EN and PN should not be initiated early in critically ill patients; instead, they should be prescribed within three to five days².
- In patients unable to tolerate full-dose EN during the first week in the ICU, the safety and benefits of initiating PN should be evaluated on a case-by-case basis³.
- Supplemental PN should be considered within 4 to 7 days if >60% of the patient’s energy and protein requirements cannot be met through the enteral route alone¹.
- In patients with severe malnutrition, early PN may be provided when EN is contraindicated, rather than leaving the patient without nutritional support¹.
- If abdominal distension or vomiting occurs during EN, potential alternative causes of these signs and symptoms should first be evaluated. If they are attributable to nutritional support therapy (NST), the infusion rate of the nutritional product should be reduced; if the problem persists, nutrition should be temporarily discontinued².
- In the event of diarrhea during EN, a careful differential diagnosis should be conducted before modifying the nutritional regimen. If diarrhea is identified as a complication of EN, the first step should be to reduce the amount of nutrition, and if necessary, alter the product composition².
- In patients at high risk of aspiration, postpyloric feeding, particularly via the jejunal route, may be considered¹.
- Blood glucose should be measured at baseline and at least every 4 hours during the first two days. Insulin therapy should be initiated when glucose levels exceed 180 mg/dL¹.
- To ensure accurate dosing in clinical practice, the most appropriate EN formula should be selected according to caloric and protein content¹.
- Enteral formulas enriched with high-dose omega-3 should not be administered routinely¹.
- In selected patients with electrolyte and fluid imbalance, renal EN formulas with lower electrolyte content and higher protein concentration (70–80 g protein/L) may be preferred over standard formulas².
- EN enriched with omega-3 fatty acids may be applied within prescribed nutritional doses¹.

**References**

1. Singer P, Blaser AR, Berger MM, Calder PC, Casaer M, Hiesmayr M, Mayer K, Montejo-Gonzalez JC, Pichard C, Preiser JC, Szczeklik W, van Zanten ARH, Bischoff SC. ESPEN practical and partially revised guideline: Clinical nutrition in the intensive care unit. Clin Nutr. 2023 Sep;42(9):1671-1689. <https://doi.org/10.1016/j.clnu.2023.07.011>.
2. Klinik Enteral Parenteral Nütrisyon Derneği (KEPAN). Enteral Beslenme (EB) Rehberi. Ankara: Klinik Enteral Parenteral Nütrisyon Derneği; 2023.
3. Cano, N., Fiaccadori, E., Tesinsky, P., Toigo, G., Druml, W., Kuhlmann, M., Mann, H., Hörl, W. H., DGEM (German Society for Nutritional Medicine), ESPEN (European Society for Parenteral and Enteral Nutrition), & others. (2021). ESPEN guideline on clinical nutrition in hospitalized patients with acute or chronic kidney disease. Clinical Nutrition, 40(4), 1644–1688. https://doi.org/10.1016/j.clnu.2021.02.025
4. Kreymann, K. G., Berger, M. M., Deutz, N. E. P., Hiesmayr, M., Jolliet, P., Kazandjiev, G., Nitenberg, G., van den Berghe, G., Wernerman, J., & Ebner, C. (2006). ESPEN Guidelines on Enteral Nutrition: Intensive care. Clinical Nutrition, 25(2), 210–223. https://doi.org/10.1016/j.clnu.2006.01.021
5. McClave, S. A., Taylor, B. E., Martindale, R. G., Warren, M. M., Johnson, D. R., Braunschweig, C., McCarthy, M. S., Davanos, E., Rice, T. W., Cresci, G. A., Gervasio, J. M., Sacks, G. S., Roberts, P. R., Compher, C., & Society of Critical Care Medicine (SCCM); American Society for Parenteral and Enteral Nutrition (ASPEN). (2021). Guidelines for the provision and assessment of nutrition support therapy in the adult critically ill patient. Journal of Parenteral and Enteral Nutrition, 45(1), 15–55. https://doi.org/10.1002/jpen.2030
