# Supplementary material for: Evaluation of guideline adherence in enteral nutrition practices among ICU patients: a point prevalence study
Source: BMJ Open. 2026 Sep 2;16(9):e120718. doi: 10.1136/bmjopen-2026-120718 (PMC13548273; doi:10.1136/bmjopen-2026-120718)
Supplement: Supplementary file 3 [file bmjopen-16-9-s002.docx]

**Table 1. Factors Influencing Adherence to Enteral Nutrition Protocols**

| Variable | **Timing of EN initiation** | | | **Method of enteral nutrition administration** | | | **Achievement of target calorie and protein targets** | | | **Management of EN-interrupting condition** | | |
| --- | --- | --- | --- | --- | --- | --- | --- | --- | --- | --- | --- | --- |
|  | **Yes** | **No** | **p** | **Yes** | **No** | **p** | **Yes** | **No** | **p** | **Yes** | **No** | **p** |
| **Sex**  *Female*  *Male* | 35  50 | 3  10 | 0.174 | 37  54 | 1  6 | 0.165 | 9  18 | 29  42 | 0.329 | 34  50 | 4  10 | 0.296 |
| **Reason for hospitalization**  *Medical*  *Surgical* | 61  24 | 12  1 | 0.102 | 67  24 | 6  1 | 0.425 | 18  9 | 55  16 | 0.2 | 63  21 | 10  4 | 0.503 |
| **Obesity**  *Yes*  *No* | 20  65 | 2  11 | 0.402 | 21  70 | 1  6 | 0.506 | 3  24 | 19  52 | 0.078 | 19  65 | 3  11 | 0.614 |
| **MV**  *Yes*  *No* | 73  12 | 12  1 | 0.456 | 78  13 | 7  0 | 0.357 | 24  3 | 61  10 | 0.494 | 74  10 | 11  3 | 0.275 |
| **Renal status**  *Normal*  *Acute kidney injury*  *Chronic kidney disease* | 67  11  7 | 7  4  2 | 0.142 | 70  13  8 | 4  2  1 | 0.492 | 25  2  0 | 49  13  9 | 0.041 | 69  9  6 | 5  6  3 | 0.001 |
| **Sepsis/Septic shock**  *Yes*  *No* | 34  51 | 6  7 | 0.139 | 37  54 | 3  4 | 0.439 | 12  15 | 28  43 | 0.322 | 31  53 | 9  5 | 0.091 |
| **EN consultation**  *Yes*  *No* | 29  56 | 1  12 | 0.046 | 30  61 | 0  7 | 0.070 | 12  15 | 18  53 | 0.058 | 28  56 | 2  12 | 0.130 |
| **EN methods**  **Continuous**  **Intermittent**  **Bolus** | 69  11  5 | 7  4  2 | 0.088 | 76  15  0 | 0  0  7 | 0.000 | 17  7  3 | 59  8  4 | 0.101 | 64  15  5 | 12  0  2 | 0.149 |
| **Complications associated with EN**  *Yes*  *No* | 28  57 | 4  9 | 0.575 | 30  61 | 2  5 | 0.587 | 9  18 | 23  48 | 0.556 | 25  59 | 7  7 | 0.119 |
| **Age (years), Median (IQR)** | 72  (61.5-79.5) | 69  (55-75) | 0.372 | 72  (59-79) | 65  (57-87) | 0.689 | 71 (54-79) | 72 (61-79) | 0.371 | 71.5 (59-79.75) | 72.5 (60-79) | 0.972 |
| **EN therapy duration** **(day), Median (IQR)** | 11  (4-23.5) | 11  (5-20) | 0.875 | 11  (4-25) | 6  (5-14) | 0.297 | 16 (4-27) | 9 (4-21) | 0.239 | 11 (4-24.75) | 7.5 (5.5-17.5) | 0.615 |
| **BMI (kg/m^2^), Median (IQR)** | 25.7  (23.3-28.1) | 23.4  (21.5-28.5) | 0.258 | 25.7  (23.1-28.3) | 22.2  (21.2-26.1) | 0.119 | 23.1 (21.1-27.7) | 26 (23.9-31.2) | 0.017 | 25.7 (23.4-28.2) | 23.4 (21.4-28.5) | 0.273 |
| **Daily calories (kcal/day),** **Median (IQR)** | 1400  (1195-1680) | 1497  (1278-1772) | 0.356 | 1410  (1200-1610) | 1881  (1254-1941) | 0.291 | 1804 (1584-2397) | 1265 (1035-1452) | 0.000 | 1405 (1200-1750) | 1440 (1016-1566) | 0.622 |
| **Daily protein (g/day), Median (IQR)** | 75.66±29.3 | 73.41±31.4 | 0.799 | 75.3±29.9 | 76.4± 24 | 0.923 | 103.9±22.9 | 64.5±23.9 | 0.000 | 76.4±29.5 | 69.1±29.5 | 0.393 |
| **Percentage of administered calories compared with target**  **Median (IQR)** | 85  (68.5-100) | 90  (74.25-100) | 0.692 | 85. 7  (68.5-100) | 97  (89.5-100) | 0.231 | 100 | 78.8  (61-89) | 0.000 | 86.4  (70-100) | 88.5 (55.5-100) | 0.836 |
| **Percentage of administered protein compared with target**  **Median (IQR)** | 83  (65.5-100) | 72.6  (54.8-100) | 0.5 | 80.8  (64.6-100) | 91  (65-100) | 0.503 | 100 | 70  (57.8-86) | 0.000 | 82  (66-100) | 88.4 (58.4-100) | 0.876 |
| **ICU length of stay (day),** **Median (IQR)** | 15  (6-26) | 14  (6.5-24.5) | 0.917 | 15  (6-26) | 7  (6-15) | 0.165 | 19  (7-30) | 13  (6-24) | 0.148 | 14  (6-26) | 16  (9.75-24) | 0.688 |
| **APACHE, Mean ± SD** | 24.24± 7.6 | 23.54±10.6 | 0.772 | 23.76±7.6 | 29.14±11.6 | 0.087 | 23.96± 9.1 | 24.2± 7.7 | 0.892 | 23.33±7.8 | 29± 8 | 0.014 |
| **SOFA, Mean ± SD** | 5.91± 3 | 9.46± 4.3 | 0.000 | 6.14± 3.2 | 9.43± 4.9 | 0.013 | 6.59± 4 | 6.3± 3.2 | 0.702 | 5.89± 3 | 9.29± 4.4 | 0.000 |
| **NUTRIC, Median (IQR)** | 6 (4.5-7) | 6 (4.5-8) | 0.706 | 6 (4-7) | 7 (5-7) | 0.878 | 6 (4-7) | 6 (5-7) | 0.249 | 6 (4-7) | 7 (5-8) | 0.029 |
| **GCS, Median (IQR)** | 8 (5-11.5) | 3 (3-8.5) | 0.009 | 8 (5-11) | 3 (3-10) | 0.171 | 9 (6-11) | 7 (4-10) | 0.229 | 8  (5.25-10) | 4.5 (3-14) | 0.276 |
| **Number of comorbidities, Median (IQR)** | 3 (1-4) | 2 (1-3.5) | 0.452 | 2 (1-4) | 3 (0-5) | 0.806 | 1 (1-3) | 3 (2-4) | 0.007 | 2 (1-4) | 3 (2-5) | 0.127 |
| **Duration of MV (days),** **Median (IQR)** | 16 (6-25) | 13.5  (4.25-18.25) | 0.445 | 16  (5.25-25.75) | 7 (6-15) | 0.261 | 17  (6-31.75) | 14 (5-24) | 0.403 | 15 (5-26) | 15.5  (6-24.75) | 0.961 |
| **Daily EN administration (h),** **Median (IQR)** | 20 (20-23) | 20 (15-23) | 0.470 | 20  (20-23) | 6 | 0.000 | 20 (20-24) | 20  (20-23) | 0.967 | 20  (20-23) | 24 (16.75-24) | 0.350 |
| APACHE: Acute Physiology and Chronic Health Evaluation, BMI: Body Mass Index, EN: Enteral Nutrition, GCS: Glasgow Coma Scale, HD: Hemodialysis, ICU: Intensive Care Unit, IQR: Interquartile Range, MV: Mechanical Ventilation, NUTRIC: Nutrition Risk in the Critically Ill, SD: Standard Deviation, SOFA: Sequential Organ Failure Assessment | | | | | | | | | | | | |
